# Exposure to Chloramine and Chloroform in Tap Water and Adverse Perinatal Outcomes in Shanghai

**DOI:** 10.3390/ijerph19116508

**Published:** 2022-05-27

**Authors:** Si-Meng Zhu, Cheng Li, Jing-Jing Xu, Han-Qiu Zhang, Yun-Fei Su, Yan-Ting Wu, He-Feng Huang

**Affiliations:** 1 International Peace Maternity and Child Health Hospital, Shanghai Jiao Tong University School of Medicine, Shanghai 200030, China; xiaguhezihua@sjtu.edu.cn (S.-M.Z.); xujingjing_0922@163.com (J.-J.X.); pathsci@outlook.com (H.-Q.Z.); f_19940624@163.com (Y.-F.S.); 2 Shanghai Key Laboratory of Embryo Original Disease, Shanghai 200030, China; 3 Obstetrics and Gynecology Hospital, Institute of Reproduction and Development, Fudan University, Shanghai 200011, China; dr.cheng.li@hotmail.com

**Keywords:** chloramine, chloroform, tap water, perinatal outcomes

## Abstract

Chloramine and chloroform are widespread in tap water due to water disinfection processes. This study was designed to explore the associations between trimester-specific exposure to chloramine and chloroform in tap water and adverse outcomes. This retrospective cohort study included 109,182 mother–infant singleton pairs in Shanghai. A logistic regression model was used to evaluate the associations of chloramine and chloroform concentrations averaged over the whole pregnancy and in each trimester with adverse outcomes, including gestational diabetes mellitus (GDM), gestational hypertensive disorders (GHD), low birthweight (LBW), small for gestational age (SGA), preterm birth (PTB) and prelabor rupture of membranes (PROM). The use of tap water with elevated chloramine levels in the first trimester was associated with GDM (OR = 1.06, 95% CI: 1.03, 1.09), while that in the second trimester was related to GHD (OR = 1.13, 95% CI: 1.09, 1.17). Chloroform levels in the third trimester were associated with LBW (OR = 1.13, 95% CI: 1.09, 1.16), PTB (OR = 1.05, 95% CI: 1.01, 1.08) and PROM (OR = 1.01, 95% CI: 1.00, 1.01). However, tap water chloroform exposure in the second trimester was negatively associated with LBW (OR = 0.95, 95% CI: 0.93, 0.98) and PTB (OR = 0.97, 95% CI: 0.94, 0.99). In conclusion, there are probably no casual associations between current tap water chloroform and chloramine levels and perinatal outcomes. However, more research focusing on the effect of chloramine and chloroform on perinatal outcomes are still warranted.

## 1. Introduction

Tap water disinfectants have been widely used since the last century to kill pathogenic organisms [1] and interrupt the spread of water-borne diseases, including cholera, typhoid and amoebic dysentery [2]. Chlorine, chlorine dioxide, chloramine, ozone and bromine are some commonly used chemicals for the disinfection of water [3]. Among them, chlorine and chloramine are the most effective disinfectants and are applied in the Shanghai public drinking water system. They interact with natural organic matter in drinking water to form chloroform [4]. Chloramine is commonly used as a secondary disinfectant and can remain active in water systems for a considerably long period [5]. In Shanghai, tap water chloroform concentrations ranged from 23.3 to 0.2 mg/L, and chloramine from 1.35 to 0.78 mg/L, in the last 5 years. Chloramine and chloroform are widely distributed in tap water, exposing humans through the daily ingestion of drinking water and inhalation and dermal absorption during showers and baths [6]. In this way, chloramine and chloroform are potential systematic health threats, leading to multiple diseases, such as bladder and brain cancers [7] and nervous and reproductive effects [8] after long-term exposure.

Pregnancy is a vulnerable period with many physiological challenges; during this time, the body is more susceptible to environmental contaminants, likely resulting in pregnancy complications, such as gestational diabetes mellitus (GDM) and gestational hypertensive disorders (GHD), and adverse birth outcomes, such as small for gestational age (SGA), low birthweight (LBW), preterm birth (PTB) and prelabor rupture of membranes (PROM). In addition to contributing to a significant proportion of neonatal deaths globally [9], these adverse outcomes also contribute to higher risks of postnatal death, childhood growth restriction and adult-onset chronic disease, including cardiovascular diseases, diabetes, cognitive impairment and psychiatric conditions [10,11,12,13]. Thus, adverse perinatal outcomes are an important issue regarding the long-term health status of infants. Concerns regarding the potential impacts of chloramine and chloroform on reproductive outcomes have been raised, as such concerns are supported by findings of increased risks of pregnancy loss, stillbirth and birth defects [8,14,15,16]. However, no studies on the effects of chloramine and chloroform on gestational complications have been published, and their association with neonatal birthweight and gestational duration remains controversial [17,18,19]. In addition, the critical periods of susceptibility to chloramine and chloroform during pregnancy have rarely been studied. Some suggested second trimester exposure could lead to adverse birth outcomes [20], while others argued that third trimester exposure was more important [21]. Therefore, we aimed to investigate whether chloramine and chloroform levels averaged over the whole pregnancy and in each trimester were associated with pregnancy complications and adverse neonatal outcomes. To our knowledge, our study is the first to investigate the association between chloramine and chloroform levels and pregnancy complications. Considering the contaminants’ concentrations were within the regulation range, this large data set allowed an assessment of low-exposed population and could provide valuable information for drinking water regulation for maternal and fetal health.

## 2. Methods

### 2.1. Study Design and Participants

Shanghai is situated in the estuary of the Yangtze River in the eastern center of China and is a major industrial and commercial city. The Obstetrics and Gynecology Hospital of Fudan University (Ob & Gyn Hospital) and International Peace Maternity and Child Health Hospital (IPMCHH), which are both situated in the center of Shanghai, are the two largest maternal and child health hospitals in Shanghai with patients from all over the city. This study was designed to assess the associations between chloramine and chloroform levels in tap water and birth outcomes. Pregnant women with singleton deliveries at IPMCHH and Ob & Gyn Hospital between 1 June 2016 and 30 October 2020 were included in the analysis. Women who lived outside of Shanghai during their pregnancy were excluded.

### 2.2. Environmental Data

Most of the tap water in Shanghai originates from Qingcaosha reservoir. According to the *Standards for Drinking Water Quality* in China, the maximum contaminant levels of chloramine and chloroform are 3 mg/L and 0.06 mg/L, respectively. Drinking water chloramine and chloroform concentrations were measured at all of the 14 water treatment plants in Shanghai by Shanghai Monitoring Center of National Urban Water Supply Quality Monitoring Network monthly. Samples were collected at the entrance of the pipe network after water treatment [22]. Then, the N,N Diethyl-p-phenylenediamine colorimetric method was performed to measure chloramine concentrations [23] and chloroform concentrations were assessed using gas chromatography with electron detection [24]. Measurements were controlled by the quality control chart method. However, only the monthly average concentrations of the contaminants of whole city were accessible, which were averaged from the data collected from the 14 water treatment plants and published by Shanghai Water Authority (Shanghai Municipal Ocean Bureau, Shanghai, China) for the entire study period. First, we matched the gestational months of each pregnancy with calendar months according to last menstrual period and the date of delivery. Then, the exposure of each gestational month was assessed based on these municipal average concentrations of chloramine and chloroform for the month. When a gestational month covered two calendar months, the data of the former months was used to present the monthly exposure levels. Accordingly, we calculated the average of monthly contaminant concentrations in tap water during each trimester to determine trimester-specific tap water chloramine/chloroform exposure levels.

### 2.3. Confounders

Maternal sociodemographic (prepregnancy body mass index (BMI), maternal age at birth, marital status, medical insurance status, occupation, residence and education level), reproductive history (including parity, previous ectopic pregnancy and abortions), mode of conception and tobacco and alcohol consumption data were obtained by in-person interviews. Data on pregnancy complications, including GDM, diabetes mellitus in pregnancy, chronic hypertension in pregnancy, GHD, gestational thyroid dysfunction, intrahepatic cholestasis of pregnancy (ICP) and newborn sex, were ascertained from the medical health records from prenatal visits and delivery. Maternal age and prepregnancy BMI were analyzed as continuous variables, while others were coded as binary variables.

Potential confounders were selected using Pearson correlations among sociodemographic characteristics, history of pregnancy and pregnancy complications for each birth outcome separately. Factors with an absolute Pearson correlation coefficient value greater than 0.02 as well as those with *p* values less than 0.05 were selected as potential confounders. After clinical considerations, the confounding factors were finally taken into the adjusted models. The directed acyclic graphs were performed to show the relationships among contaminants, confounders and perinatal outcomes.

### 2.4. Perinatal Outcomes

The outcomes of interest in this study were GDM, GHD, LBW, SGA, PTB and PROM. GDM was defined according to diagnostic criteria proposed by the American Diabetes Association [25]. GHD was diagnosed according to the American College of Obstetricians and Gynecologists Practice Bulletin [26]. Gestational age was calculated according to the date of the last menstrual period, and PTB was defined as infants born before 37 gestational weeks. SGA was defined as an infant with birthweight below the 10th percentile for his/her gestational age and sex [27]. LBW was defined as a birthweight of less than 2500 g. PROM was defined as membrane rupture occurring before the onset of labor. The diagnosis relied on visualization of amniotic fluid leakage from the cervical canal and pooling in the vagina, confirmed by an alkaline pH according to a nitrazine paper test and the presence of a ferning pattern under microscopy [28].

### 2.5. Statistical Analysis

After adjustment for potential covariates, we developed a logistic regression model with a spline function to calculate the odds ratios (ORs) and their 95% confidential intervals (CIs) for the associations of trimester-specific chloramine and chloroform levels in tap water with dichotomous perinatal outcomes, including GDM, GHD, LBW, SGA, PTB and PROM. In the model, trimester-specific chloramine and chloroform concentrations were treated as continuous variables. We calculated the monthly attack rates of SGA, PTB and PROM as the percentages of these birth outcomes among all singleton live births. Then, we plotted the trends of the monthly attack rates with a generalized linear regression model with a spline smoothing function. Additionally, the trends of monochloramine and chloroform were shown to visualize their general association with adverse perinatal outcomes. As the data of monochloramine and chloroform were calculated by using the data in the former month if a gestational month covered two calendar months, sensitivity analysis was also preformed using the data in the latter month to confirm their associations with adverse perinatal outcomes. All analyses included only live births and singleton pregnancies to reduce potential bias due to stillbirths and multifetal pregnancies. All analyses were performed using R software (version 4.04, R Foundation for Statistical Computing, Vienna, Austria).

## 3. Results

### 3.1. Characteristics of the Mother–Infant Pairs

A total of 109,182 mother- infant pairs in Ob & Gyn Hospital and IPMCHH during June, 2016 to October, 2020 were included in the study. The majority of infants were born to mothers who were Han nationality (98.6), had completed college education (84.4%), were married (99.1%), had medical insurance (71.8%) and were nulliparous (60.3%), with a mean age of 31.01 years and a mean BMI of 21.23. Only 0.4% of mothers reported cigarette consumption, and 0.9% reported alcohol consumption (Table 1). For pregnancy women, GDM had the highest morbidity (13.9%) among the pregnancy complications, followed by gestational hypertensive disorder (6.2%). Among all singleton live births, 3911 (3.6%) were LBW, 2369 (2.2%) were SGA, 6240 (5.7%) were PTB, 22956 (21.0%) were PROM. About half of the newborn infants were male (51.7%) and vaginal birth (52.4%) (Table 2).

We performed a Pearson correlation analysis to filter potential confounders. Eight variables were associated with GDM, as shown in Figure S1, and Figure S2 shows the eight characteristics associated with GHD. Five variables were found to be positively related to LBW (Figure S3), and three variables were relevant to SGA (Figure S4). Six variables were selected for PTB (Figure S5) and seven for PROM (Figure S6). All of the selected confounders were added to the multivariable logistic regression model after clinical considerations.

### 3.2. Risk of Adverse Perinatal Outcomes

In our study, we analyzed the association between tap water chloramine and chloroform and adverse perinatal outcomes, including GDM, GHD, LBW, SGA, PTB and PROM, by logistic regression (Table 3).

After adjustment for confounders, each unit increase in monochloramine in the first trimester was associated with an increased risk of GDM (OR = 1.06, 95% CI: 1.03, 1.09). Additionally, we observed a positive association between monochloramine levels in the second trimester and GHD (OR = 1.13, 95% CI: 1.09, 1.17). However, the chloroform concentration in tap water was not related to any pregnancy complications.

After adjustment for confounders, elevated tap water chloramine concentrations during the third trimester of pregnancy were associated with an increased risk of LBW (OR = 1.06, 95% CI: 1.01, 1.11). Similarly, use of tap water with increased chloroform in the first trimester was associated with a risk of LBW (OR = 1.05, 95% CI: 1.03, 1.07) as well as in third trimesters (OR = 1.13, 95% CI: 1.09, 1.16), while that in the second trimester showed a negative association with LBW (OR = 0.95, 95% CI: 0.93, 0.98). Additionally, increasing chloroform concentrations during the entire pregnancy were associated with an increased risk of LBW (OR = 1.05, 95% CI: 1.02, 1.08). As for SGA, only a slight negative association between monochloramine in the second trimester was exhibited. We observed a slight increase in the odds of PTB with second trimester chloramine (OR = 1.04, 95% CI: 1.00, 1.08), and the risk of PTB increased significantly with increasing tap water chloroform concentrations in the first (OR = 1.04, 95% CI: 1.02, 1.06) and third (OR = 1.05, 95% CI: 1.01, 1.08) trimesters instead. However, monochloramine in the first trimester (OR = 0.95, 95% CI: 0.92, 0.99) and chloroform concentrations in the second trimester (OR = 0.97, 95% CI: 0.94, 0.99) showed negative associations with PTB. In addition, tap water chloramine (OR = 1.10, 95% CI: 1.03, 1.19) and chloroform (OR = 1.07, 95% CI: 1.03, 1.11) during the entire pregnancy were significantly related to PTB. We also observed a significant association between the risk of PROM and tap water chloramine concentration in the first (OR = 0.95, 95% CI: 0.93, 0.97) and third (OR = 1.04, 95% CI: 1.01, 1.07) trimesters, while they indicated contradicted relationships. Additionally, a weak elevation in the odds of PROM with third trimester chloroform levels (OR = 1.01, 95% CI: 1.00, 1.01) was observed. Similar to PTB, PROM was positively associated with chloramine (OR = 1.07, 95% CI: 1.03, 1.12) and chloroform (OR = 1.02, 95% CI: 1.01, 1.03) throughout the entire pregnancy.

Subsequently, the analyses were stratified according to fetal sex. In both male and female infants, the effects of monochloramine on perinatal outcomes were similar to those in the total population (Tables S1 and S2). While in male fetuses, the risks of SGA were positively associated with chloroform exposure in the third trimester.

**Figure 1:**
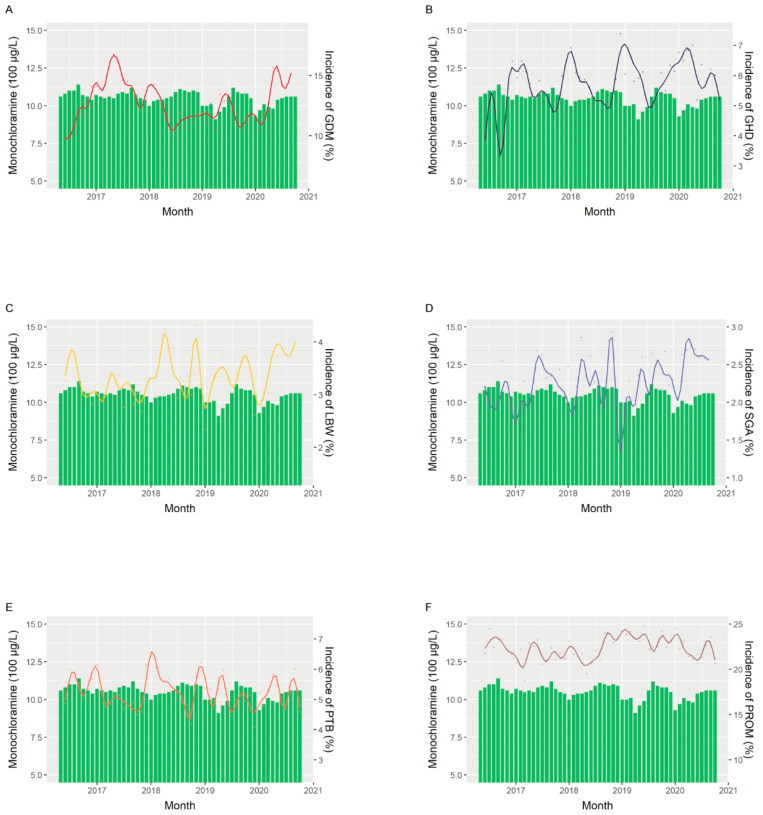
Trends of monochloramine levels and monthly attack rates of perinatal outcomes. Histogram shows the trends of monochloramine levels, and fitted spline curve shows the monthly attack rates of (**A**) gestational diabetes mellitus (GDM) and (**B**) gestational hypertension disorders (GHD), (**C**) low birth weight (LBW), (**D**) small for gestational age (SGA), (**E**) preterm birth (PTB) and (**F**) prelabor rupture of membranes (PROM).

**Figure 2:**
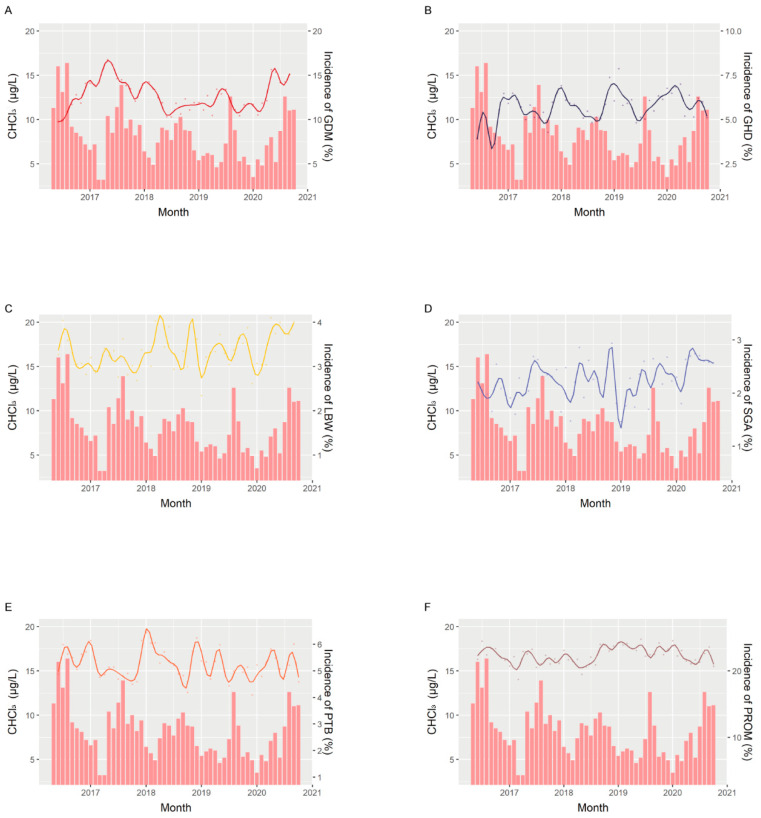
Trends of chloroform levels and monthly attack rates of perinatal outcomes. Histogram shows the trends of chloroform levels, and fitted spline curve shows the monthly attack rates of (**A**) gestational diabetes mellitus (GDM) and (**B**) gestational hypertension disorders (GHD), (**C**) low birth weight (LBW), (**D**) small for gestational age (SGA), (**E**) preterm birth (PTB) and (**F**) prelabor rupture of membranes (PROM).

The chloramine and chloroform trends and fitted lines of the attack rates of perinatal outcomes are shown in Figure 1 and Figure 2. The spline curve for GDM showed a trend similar to that of monochloramine concentration, with a lag of approximately 9 months, while the trend of GHD followed that of monochloramine concentration, with a 6-month lag, which is consistent with our findings in Table 3 (Figure 1A,B). We observed similar trends of LBW and PROM with monochloramine, with an approximately 3-month lag, which indicates the relationship between third trimester exposure and birth outcomes (Figure 1C,F). For the trend of chloroform, the spline curves of LBW, PTB and PROM were similar, with an approximately 3-month lag, suggesting the potential effect of chloroform concentration in the third trimester on birth outcomes (Figure 2C,E,F).

## 4. Discussion

In recent years, disinfectants and their by-products have received increasing attention due to health concerns. In this study, we examined the relationship between tap water chloramine/chloroform concentrations and several adverse perinatal outcomes in Shanghai, China. By analyzing all live singleton births, our results showed that chloramine levels in the first and second trimesters was associated with GDM and GHD, respectively. For birth outcomes, chloramine and chloroform concentrations during the third trimester were associated with increased risks of LBW, PTB and PROM, while the relationships between birth outcomes and contaminants in the first and second trimesters were inconsistent. However, there was no evidence of any association of chloramine and chloroform in either trimester with SGA. To our knowledge, this is the first study to examine the association between water disinfectants and their by-products throughout pregnancy with pregnancy complications.

Monochloramine, a long-lasting drinking water disinfectant, is believed to be safe and appropriate and has been approved by the United States Environmental Protection Agency (EPA). In the 1980s–1990s, several studies raised concerns about its health risks. In a study by Lubbers et al. [29], there were no detectable adverse effects of chloramine at a dose of 5 mg/L on physical condition, while another study found that an increased concentration of chloramine was related to an outbreak of erythropoietin resistance in 1996 in London, UK [30]. However, the reproductive toxicity of chloramine in humans has rarely been studied, although several animal studies have been performed. A study showed that monochloramine exposure did not harm female fertility, reproductive organs, pup weight or litter size in rats [31]. Additionally, neither teratogenic nor embryotoxic effects of monochloramine have been found in animal studies [32]. To our knowledge, there have been no recent studies on the health risks of chloramine. Therefore, we studied this issue and found that chloramine levels in the third trimester were related to GDM, GHD, LBW and PROM, which is inconsistent with previous animal studies. Meanwhile, tap water chloramine in early pregnancy showed slightly negative associations with LBW, PTB and PROM, implying the different role of chloramine in different trimesters. Due to its large sample size, our study potentially provides new insight into the safety of chloramine use in drinking water disinfection, especially for pregnant women and neonatal infants, but additional epidemiological studies as well as mechanistic investigations are needed to clarify the effect of chloramine exposure on human health. Unlike chloramine, the health risks of chloroform, the dominant species of disinfection by-products (DBPs), have been well studied in the last two decades, as summarized in Table S3. In our study, we found that maternal chloroform concentrations in the third trimester were associated with LBW, PTB and PROM, while exposure during the first trimester was associated with LBW and PTB. In supporting our findings, three studies found chloroform exposure during the entire pregnancy was considerably associated with LBW [17,33,34]. Conversely, we observed a negative relationship between the tap water chloroform levels in the second trimester and the risks of LBW and PTB; two studies from China indicated a lack of association between chloroform exposure during pregnancy and LBW and PTB [21,35]. Two other studies from Spain and Lithuania [36,37] came to a similar conclusion. Our study only showed that monochloramine in the second trimester was negatively associated with SGA, and chloroform was not related to SGA, consistent with several epidemiological studies [36,37,38]. However, several studies reported a positive association of SGA with chloroform exposure [19,20,21,38]. During the third trimester, the fetus develops at the highest speed; thus contaminant exposure in the third trimester, rather than the first or second trimesters, has a more direct effect on fetal growth. This could partly explain the inconsistent results found in the first and second trimesters of LBW, and emphasize the importance of third trimester exposure. However, how chloroform impacts gestational duration remains unclear and more epidemiological and mechanism studies are warranted. The effect of chloroform on PROM has rarely been studied since it might be regarded as a result of acute exposure rather than long-term exposure. Our study may provide new insights into the cumulative effect of environmental pollution on PROM.

Different measurement methods of maternal exposure may largely contribute to the discrepancies between studies. Most previous studies used data from water distribution networks to estimate individual exposure by geocoding residential addresses, while two studies measured blood chloroform concentrations to reflect integrative contaminant exposure more precisely. In this study, we assessed the overall chloroform and chloramine levels of the whole city using the average contaminant concentrations reported from various water networks over a 5-year period. In addition, regional and temporal variability in chloroform concentration may partly explain the differences in these study results. In Shanghai, the mean tap water chloroform concentration was 8.17 μg/L between 2016 and 2020. It was among the lowest levels in all previous studies. In addition, the inconsistences among studies may be partly explained by the variability in covariate selection. Most of the studies selected their covariates based on a literature review, and several studies lacked data on pregnancy complications, including GDM and GHD, which may play important roles in fetal development. We used statistical methods as well as clinical considerations to select confounders for a more proper statistical model. However, some important factors are missing like dietary nutrition and physical activity, which may contribute to bias of perinatal outcomes.

Toxicological studies have demonstrated that oxidative stress may play a key role in the relationships of chloroform and chloramine exposure with complications and fetal growth. Studies have shown that blood chloroform concentrations in pregnant women have a dose-response relationship with urinary oxidative biomarkers, such as 8-hydroxy-2-deoxyguanosine [39]. In an in vitro study, Beddows et al. found that chloroform induced oxidative stress via glutathione depletion, causing lipid peroxidation and DNA strand breakage. Epigenetic alterations are also involved in this process [40]; hypomethylation occurred and gene expression was modified [41]. The reduction-oxidative status and disrupted methylation could alter in the uterus environment and affect fetal development or lead to pregnancy complications eventually [42,43]. However, mechanisms underlying the effect of chloramine and chloroform on perinatal outcomes are not well elucidated yet and more studies are needed.

The vulnerable window regarding contaminants during pregnancy has been debated. As most of the pregnancy complications had already developed by the third trimester, our study reasonably suggests that pollutant exposure in the first and second trimesters may be essential considering placentation and maternal adaptation. Regarding fetal growth, some research has proposed that exposure in the third trimester is associated with the highest risk because blood flow increases in this period to meet fetal growth, thus exposing the fetus to more contaminants [44]. Our findings support the hypothesis that the third trimester may be a critical period of susceptibility to contaminants in terms of fetal development, which is consistent with several studies that have investigated internal and external exposure levels. However, the effect of exposure in the first and second trimesters remains controversial and more studies focusing on this issue are urgently needed.

Chloramine and chloroform exposure may have indirect effects on birthweight. Our study found a positive association between monochloramine and GHD; the latter is a major contributor to LBW due to placental dysfunction [45]. Therefore, we assumed a mediating effect of GHD on the relationship between monochloramine exposure and LBW. In addition, monochloramine and chloroform concentrations were positively associated with LBW, while no associations were found for SGA, suggesting that decreased birthweight could have been caused by a shorter gestational duration. Thus, PTB may be another important mediator for LBW. However, chloroform exposure in the third trimester was found to be related to SGA in male fetuses, indicating male fetuses maybe more susceptible, which is consistent with previous studies [46,47,48].

Our study has several strengths, including a prolonged study period spanning 5 years, a large sample size, a comprehensive number of potential confounders and trimester-specific exposure data. Importantly, this study investigated pregnancy complications associated with exposure to water disinfectants and their by-products.

There are also some limitations. First, we lacked individual-specific exposure data, such as internal exposure biomarkers or estimated daily uptakes, considering the variability in water use. Additionally, we only provided evidence that tap water chloramine and chloroform concentrations were associated with perinatal outcomes, but it is hard to tell whether drinking water was responsible for the associations. Additionally, when assigning the contaminants’ concentrations to gestational months, data in the former month were applied if a gestational month covered two calendar months. Considering the potential bias, we performed a sensitivity analysis using the data in the latter month, and found similar results (Table S4). In addition, although there were spatial variations in personal exposure among residents, we lacked data on contaminant concentrations in different water treatment plants and performed our study mainly based on temporal exposure differences. In other words, we analyzed the relationship between the overall levels of chloramine and chloroform in Shanghai and perinatal outcomes instead of the effect of maternal individual exposure. Moreover, considering the deficit in exposure assessment of our study and the small deviation from 1 in ORs, we could not prove a causal relationship between the contaminants in tap water and perinatal outcomes.

## 5. Conclusions

In our study, both positive and negative associations between tap water chloramine and chloroform levels in different trimesters and perinatal outcomes were found, indicating there is probably no casual association between the current level of contaminants and adverse pregnancy outcomes. However, more research is still warranted on the potential effect of chloramine and chloroform on perinatal outcomes with more accurate exposure measurements due to the potential risks.

## Figures and Tables

**Table 1:** Maternal characteristics of all pregnant women.

	ALL	GDM	GHD	LBW	SGA	Preterm	PROM
	(*n* = 109,182)	(*n* = 15,183)	(*n* = 6791)	(*n* = 3911)	(*n* = 2369)	(*n* = 6240)	(*n* = 26,618)
	No. (%)	No. (%)	No. (%)	No. (%)	No. (%)	No. (%)	No. (%)
**Maternal sociodemographic characteristics**							
**Age, mean ± SD, years**	31.01 ± 4.06	32.53 ± 4.26	31.77 ± 4.40	31.48 ± 4.36	30.58 ± 4.00	31.73 ± 4.37	30.758 ± 3.896
**Pre-gestational BMI, mean ± SD, kg/m^2^**	21.23 ± 2.81	22.31 ± 3.18	23.06 ± 3.57	21.31 ± 3.14	20.408 ± 2.61	21.67 ± 3.14	21.191 ± 2.746
**Marriage**							
Married	108,202 (99.1)	15,010 (98.9)	6713 (98.9)	3883 (99.3)	2356 (99.45)	6198 (99.3)	26,451 (99.4)
Single	979 (0.90)	173 (1.1)	78 (1.2)	28 (0.7)	13 (0.55)	42 (0.7)	167 (0.6)
**Medical insurance**							
Yes	78,365 (71.8)	10,814 (71.2)	4990 (73.5)	2830 (72.4)	1687 (71.21)	4478 (71.8)	19,545 (73.4)
No	30,816 (28.2)	4369 (28.8)	1801 (26.5)	1081 (27.6)	682 (28.79)	1762 (28.2)	7073 (26.6)
**Occupation**							
Employed	101,825 (93.3)	13,971 (92.0)	6235 (91.8)	3565 (91.1)	2175 (91.81)	5726 (91.8)	25,151 (94.5)
Self-employed	3015 (2.8)	503 (3.3)	216 (3.2)	138 (3.5)	74 (3.12)	209 (3.4)	625 (2.4)
Unemployed	4341 (4.0)	709 (4.7)	340 (5.0)	208 (5.3)	120 (5.07)	305 (4.9)	842 (3.2)
**Ethnicity**							
Han	107,694 (98.6)	14,959 (98.5)	6685 (98.4)	3862 (98.8)	2332 (98.44)	6166 (98.8)	26,331 (98.9)
Minority	1359 (1.2)	213 (1.4)	92 (1.4)	43 (1.1)	33 (1.39)	66 (1.1)	258 (1.0)
Foreign	128 (0.1)	11 (0.1)	14 (0.2)	6 (0.2)	4 (0.17)	8 (0.1)	29 (0.1)
**Education attainment**							
High school or lower	13,152 (15.6)	2041 (17.4)	1166 (23.1)	548 (19.2)	282 (16.25)	869 (18.8)	3067 (15.0)
College	53,869 (63.9)	7617 (65.1)	3173 (62.9)	1792 (62.8)	1096 (63.2)	2918 (63.2)	12,880 (63.1)
Master or above	17,229 (20.5)	2049 (17.5)	707 (14.0)	513 (18.0)	357 (20.6)	828 (17.9)	4463 (21.9)
**Smoking during pregnancy**							
No	86,167 (99.6)	11,870 (99.5)	5208 (99.4)	3006 (99.7)	1805 (99.8)	4799 (99.6)	20,909 (99.6)
Yes	350 (0.4)	60 (0.5)	32 (0.6)	10 (0.3)	4 (0.2)	20 (0.42)	84 (0.4)
**Alcohol drinking during pregnancy**							
No	85,716 (99.1)	11,809 (99.0)	5199 (99.2)	2997 (99.4)	1799 (99.5)	4773 (99.05)	20,778 (99.0)
Yes	802 (0.9)	121 (1.0)	41 (0.8)	19 (0.6)	10 (0.6)	46 (1.0)	215 (1.)
**History of reproduction**							
**parity**							
0	65,812 (60.3)	8594 (56.6)	4432 (65.3)	2496 (63.8)	1646 (69.5)	3664 (58.7)	17,713 (66.6)
1	36,739 (33.7)	5506 (36.3)	2026 (29.8)	1198 (30.6)	632 (26.7)	2124 (34.0)	7891 (29.7)
≥2	6629 (6.1)	1083 (7.1)	333 (4.9)	217 (5.6)	91 (3.8)	451 (7.2)	1013 (3.8)
**Number of previous abortions**							
0	57,292 (63.5)	7206 (57.6)	3409 (62.5)	2045 (63.9)	1329 (70.1)	3121 (61.0)	14,246 (65.3)
1–2	29,753 (33.0)	4669 (37.4)	1823 (33.4)	1022 (31.9)	516 (27.2)	1749 (34.2)	6920 (31.7)
≥3	3192 (3.5)	627 (5.0)	221 (4.1)	136 (4.3)	52 (2.7)	244 (4.8)	664 (3.0)
**Previous ectopic pregnancy**							
No	88,270 (97.8)	12,155 (97.2)	5302 (97.2)	3142 (98.1)	1870 (98.6)	4989 (97.6)	21,388 (98.0)
Yes	1967 (2.2)	347 (2.8)	151 (2.8)	61 (1.9)	27 (1.4)	125 (2.4)	442 (2.0)

Note: GDM, gestational diabetes mellitus; GHD, gestational hypertensive disorders; LBW, low birthweight; SGA, small for gestational age; PROM, prelabor rupture of membranes; BMI, body mass index; SD, standard deviation.

**Table 2:** Gestational complications of all pregnant women.

	ALL	GDM	GHD	LBW	SGA	Preterm	PROM
	(*n* = 109,182)	(*n* = 15,183)	(*n* = 6791)	(*n* = 3911)	(*n* = 2369)	(*n* = 6240)	(*n* = 26,618)
	No. (%)	No. (%)	No. (%)	No. (%)	No. (%)	No. (%)	No. (%)
**Newborn gender**							
Male	56,449 (51.7)	7871 (51.8)	3482 (51.3)	1859 (47.5)	1126 (47.5)	3539 (56.7)	14,018 (52.7)
Female	52,732 (48.3)	7312 (48.2)	3309 (48.7)	2052 (52.5)	1243 (52.5)	2701 (43.3)	12,600 (47.3)
**Gestational diabetes mellitus**							
No	93,998 (86.1)	-	5404 (79.6)	3190 (81.6)	2060 (87.0)	5083 (81.5)	23,162 (87.0)
Yes	15,183 (13.9)	-	1387 (20.4)	721 (18.4)	309 (13.0)	1157 (18.5)	3456 (13.0)
**Diabetes mellitus in pregnancy**							
No	108,544 (99.4)	-	6658 (98.0)	3870 (99.0)	2358 (99.5)	6162 (98.8)	26,487 (99.5)
Yes	637 (0.6)	-	133 (2.0)	41 (1.1)	11 (0.5)	78 (1.3)	131 (0.5)
**Chronic hypertension in pregnancy**							
No	107,808 (98.7)	14,823 (97.6)	-	3766 (96.3)	2321 (98.0)	6047 (96.9)	26,399 (99.2)
Yes	1373 (1.3)	360 (2.4)	-	145 (3.7)	48 (2.0)	193 (3.1)	219 (0.8)
**Gestational hypertensive disorder**							
No	102,390 (93.8)	13,796 (90.9)	-	3040 (77.7)	1963 (82.9)	5267 (84.4)	25,472 (95.7)
Gestational hypertension	3023 (2.8)	632 (4.2)	-	134 (3.4)	88 (3.7)	183 (2.9)	644 (2.4)
Preeclampsia	2395 (2.2)	495 (3.3)	-	191 (4.9)	110 (4.6)	198 (3.2)	375 (1.4)
Sever preeclampsia	1373 (1.3)	260 (1.7)	-	546 (14.0)	208 (8.8)	592 (9.5)	127 (0.5)
**Intrahepatic cholestasis of pregnancy**							
No	108,345 (99.2)	15,064 (99.2)	6684 (98.4)	3838 (98.1)	2344 (98.9)	6088 (97.6)	26,517 (99.6)
Yes	836 (0.8)	119 (0.8)	107 (1.6)	73 (1.9)	25 (1.1)	152 (2.4)	101 (0.4)
**Gestational thyroid dysfunction**							
No	101,419 (92.9)	14,069 (92.7)	6252 (92.1)	3630 (92.8)	2195 (92.7)	5809 (93.1)	24,790 (93.1)
Hyperthyroidism	1102 (1.0)	163 (1.1)	96 (1.4)	52 (1.3)	30 (1.3)	78 (1.3)	283 (1.1)
Hypothyroidism	6660 (6.1)	951 (6.3)	443 (6.5)	229 (5.9)	144 (6.1)	353 (5.7)	1545 (5.8)
**Mode of delivery**							
Vaginal	57,161 (52.4)	6852 (45.1)	2143 (31.6)	1436 (36.7)	1139 (48.1)	2648 (42.4)	17,918 (67.3)
Cesarean section	47,569 (43.6)	7785 (51.3)	4382 (64.5)	2398 (61.3)	1118 (47.2)	3463 (55.5)	7114 (26.7)
Instrumental	4451 (4.1)	546 (3.6)	266 (3.9)	77 (2.0)	112 (4.7)	129 (2.1)	1586 (6.0)
**Assisted reproductive technology**							
No	101,731 (93.2)	13,585 (89.5)	5966 (87.9)	3595 (91.9)	2207 (93.2)	5713 (91.7)	25,039 (94.1)
Yes	7439 (6.8)	1598 (10.5)	823 (12.1)	315 (8.1)	161 (6.8)	516 (8.3)	1576 (5.9)

Note: GDM, gestational diabetes mellitus; GHD, gestational hypertensive disorders; LBW, low birthweight; SGA, small for gestational age; PTB, preterm birth; PROM, prelabor rupture of membranes; SD, standard deviation. The sum does not necessarily equal the sample size for all the variables due to missing data.

**Table 3:** Odds ratio of perinatal outcomes with increase of water pollutant (per unit). All analyses were adjusted for selected confounders.

	GDM OR (95% CI)	GHD OR (95% CI)	LBW OR (95% CI)	SGA OR (95% CI)	PTB OR (95% CI)	PROM OR (95% CI)
**Monochloramine (100μg/L)**						
T1	1.06 (1.03, 1.09)	0.99 (0.95, 1.03)	0.95 (0.92, 0.99)	1.00 (0.95, 1.05)	0.95 (0.92, 0.99)	0.95 (0.93, 0.97)
T2	1.00 (0.98, 1.03)	1.13 (1.09, 1.17)	0.98 (0.94, 1.02)	0.94 (0.89, 0.99)	1.04 (1.00, 1.08)	1.02 (0.99, 1.04)
T3	-	-	1.06 (1.01, 1.11)	1.03 (0.97, 1.09)	1.02 (0.98, 1.06)	1.04 (1.01, 1.07)
All	0.93 (0.88, 0.98)	1.22 (1.13, 1.31)	1.02 (0.94, 1.10)	0.95 (0.85, 1.05)	1.1 (1.03, 1.19)	1.07 (1.03, 1.12)
**CHCl_3_ (μg/L)**						
T1	1.00 (0.98, 1.01)	1.00 (0.98, 1.02)	1.05 (1.03, 1.07)	0.99 (0.96, 1.02)	1.04 (1.02, 1.07)	1.00 (0.99, 1.00)
T2	1.01 (0.99, 1.03)	1.02 (0.99, 1.05)	0.95 (0.93, 0.98)	1.00 (0.96, 1.03)	0.97 (0.94, 0.99)	1.01 (1.00, 1.01)
T3	-	-	1.13 (1.09, 1.16)	1.00 (0.96, 1.05)	1.05 (1.01, 1.08)	1.01 (1.00, 1.01)
All	1.01 (0.98, 1.04)	1.04 (1.00, 1.08)	1.05 (1.02, 1.08)	0.97 (0.92, 1.02)	1.07 (1.03, 1.11)	1.02 (1.01, 1.03)

Note: GDM, gestational diabetes mellitus; GHD, gestational hypertensive disorders; LBW, low birthweight; SGA, small for gestational age; PTB, preterm birth; PROM, prelabor rupture of membranes; OR, odds ratio; CI, confidential interval.

## Data Availability

The data presented in this study are available upon request from the corresponding author.

## Supplementary Materials

The following supporting information can be downloaded at: https://www.mdpi.com/article/10.3390/ijerph19116508/s1, Supplementary Table S1: Odds ratio of perinatal outcomes with increase of water pollutant (per unit) in male infants. Supplementary Table S2: Odds ratio of perinatal outcomes with increase of water pollutant (per unit) in female infants. Supplementary Table S3: Summary of publications on the association between water quality and birthweight. Table S4: Sensitivity analysis for odds ratio of perinatal outcomes with increase of water pollutant. Supplementary Figure S1: Pearson correlation between each feature and GDM. Bar plots from left to right are absolute values from high to low. Supplementary Figure S2: Pearson correlation between each feature and GHD. Bar plots from left to right are absolute values from high to low. Supplementary Figure S3: Pearson correlation coefficients between each feature and LBW. Bar plots from left to right are absolute values from high to low. Supplementary Figure S4: Pearson correlation coefficients between each feature and SGA. Bar plots from left to right are absolute values from high to low. Supplementary Figure S5: Pearson correlation coefficients between each feature and PTB. Bar plots from left to right are absolute values from high to low. Supplementary Figure S6: Pearson correlation coefficients between each feature and PROM. Bar plots from left to right are absolute values from high to low. Supplementary Figure S7: Directed acyclic graph showing the association among tap water contaminants, perinatal outcomes and confounders.
