# Supplementary material for: Exposure to Chloramine and Chloroform in Tap Water and Adverse Perinatal Outcomes in Shanghai
Source: Int J Environ Res Public Health. 2022 May 27;19(11):6508. doi: 10.3390/ijerph19116508 (PMC9180198; doi:10.3390/ijerph19116508)
Supplement: Supplementary file 1 [file ijerph-19-06508-s001.zip › Figure s7.pdf]

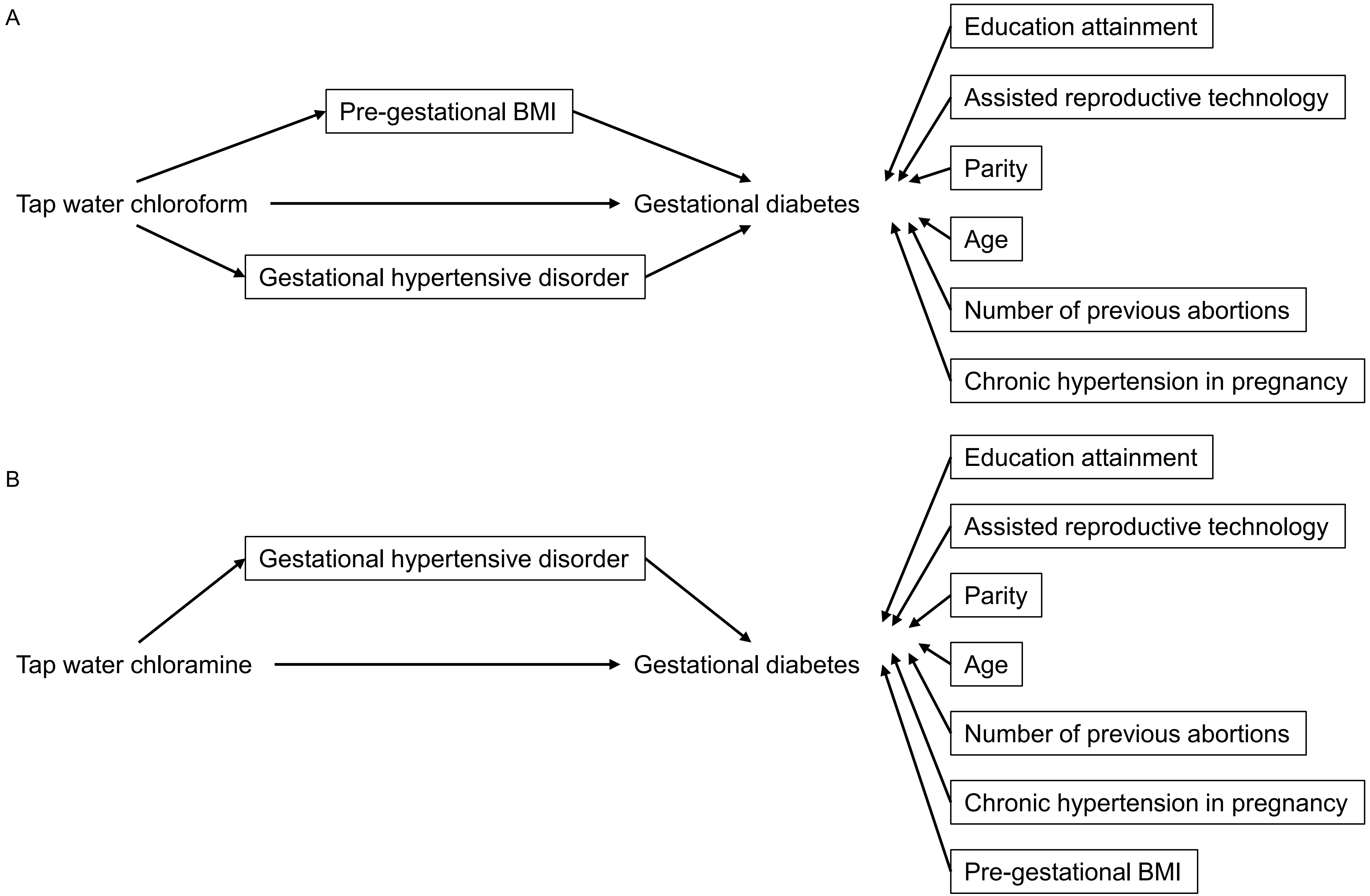

1. Directed acyclic graph showed the association among tap water contaminants, gestational diabetes and confounders: A, tap water chloroform; B, tap water chloramine

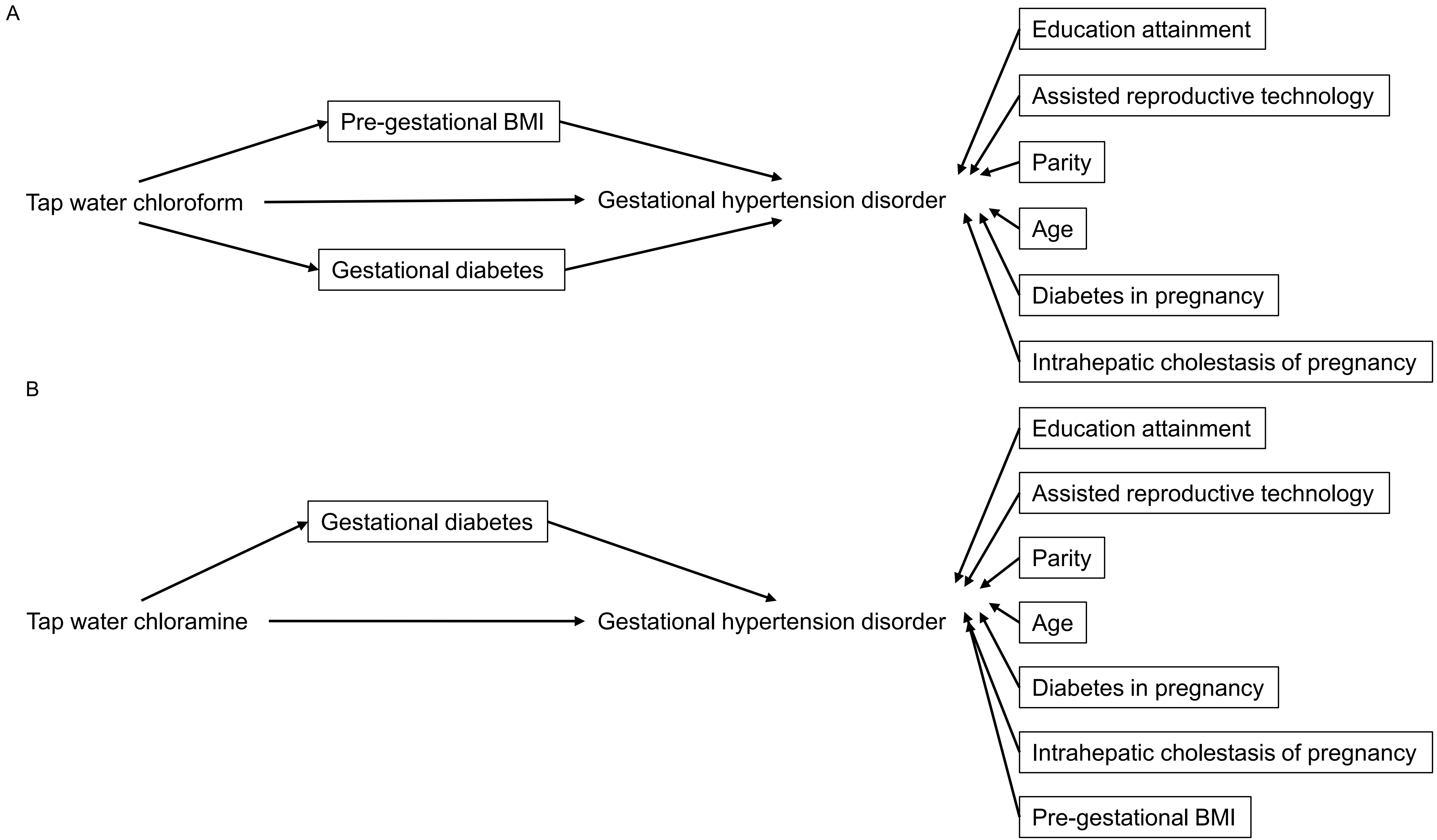

2. Directed acyclic graph showed the association among tap water contaminants, gestational diabetes and confounders: A, tap water chloroform; B, tap water chloramine

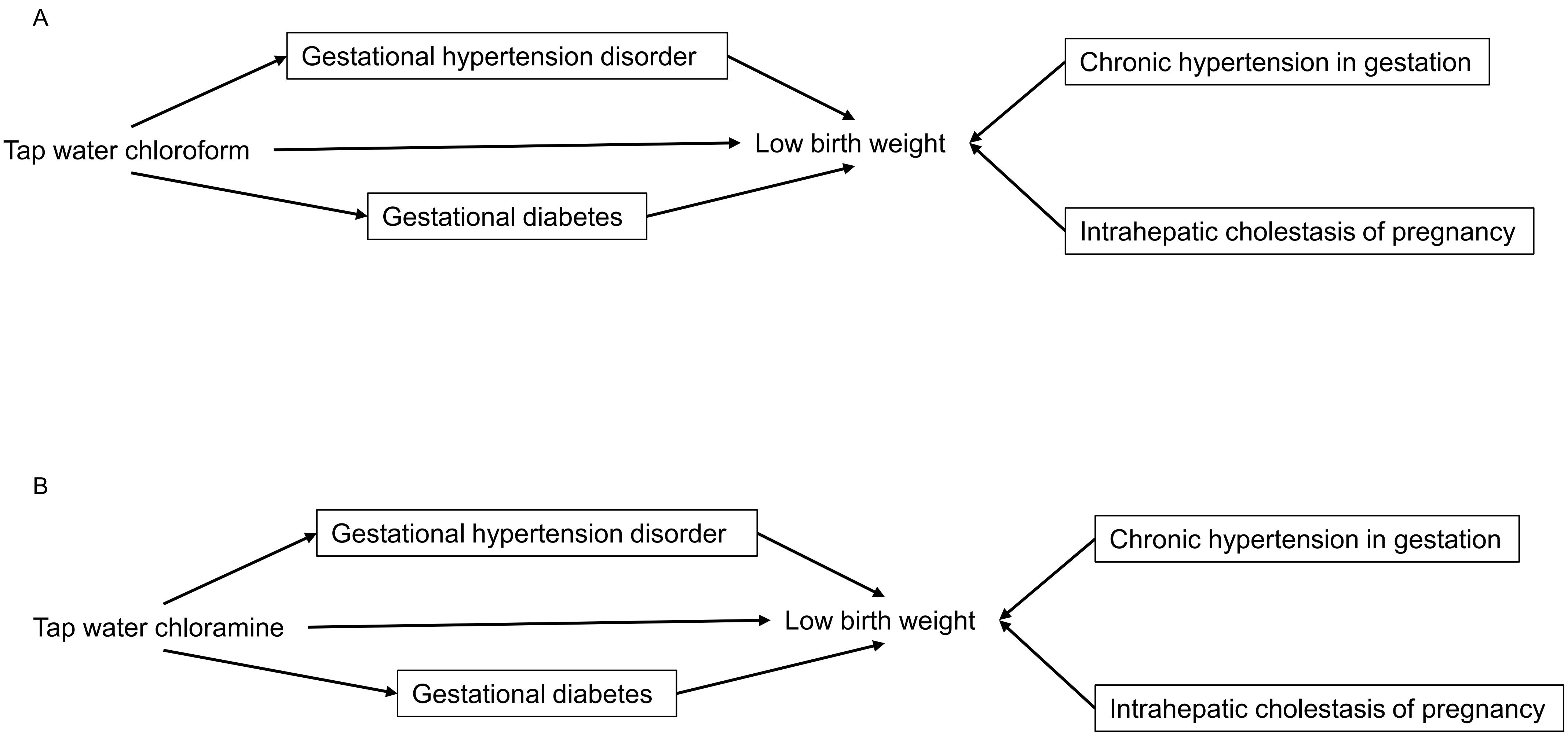

3. Directed acyclic graph showed the association among tap water contaminants, low birth weight and confounders: A, tap water chloroform; B, tap water chloramine

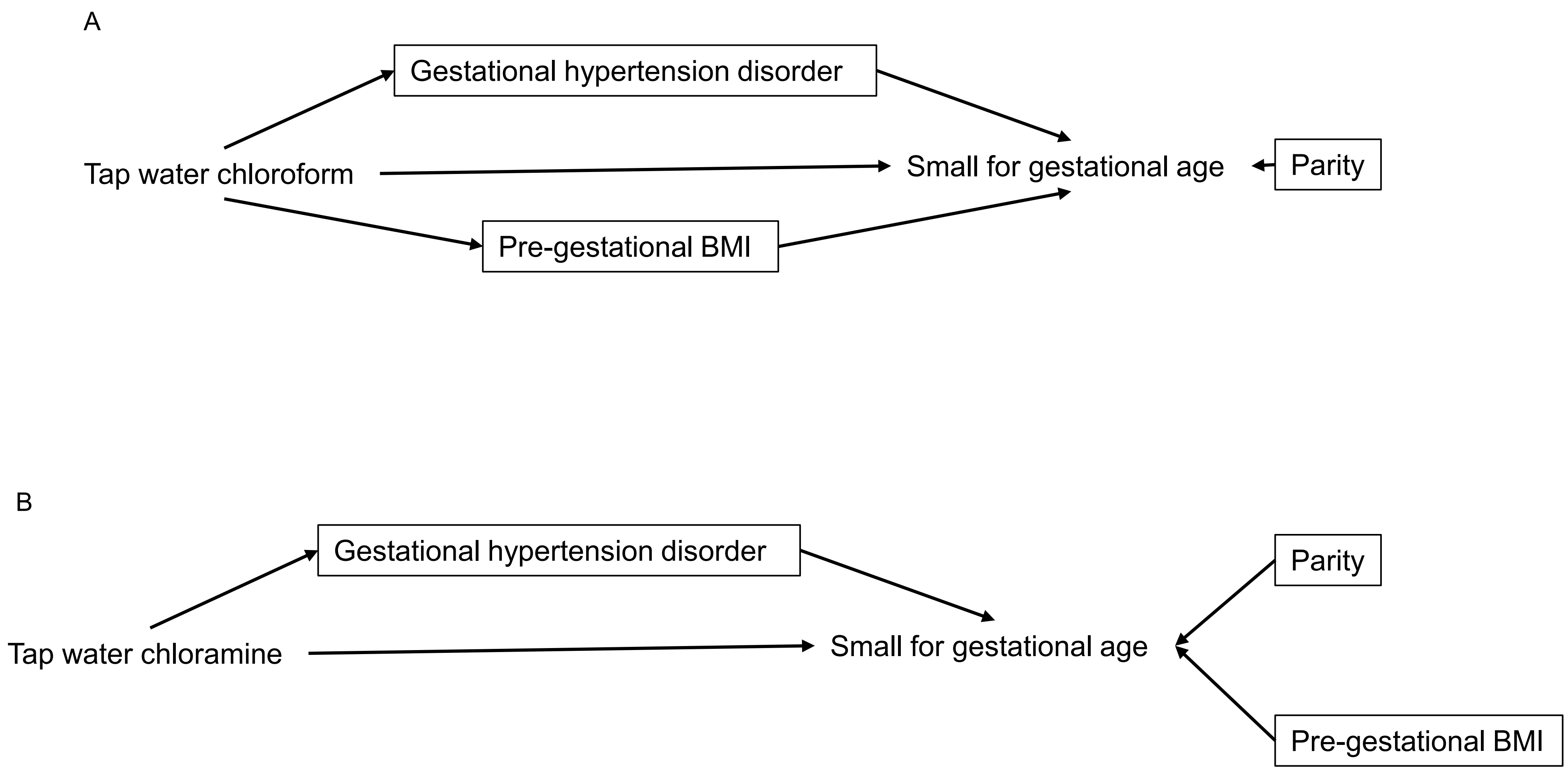

4. Directed acyclic graph showed the association among tap water contaminants, small for gestational age and confounders: A, tap water chloroform; B, tap water chloramine

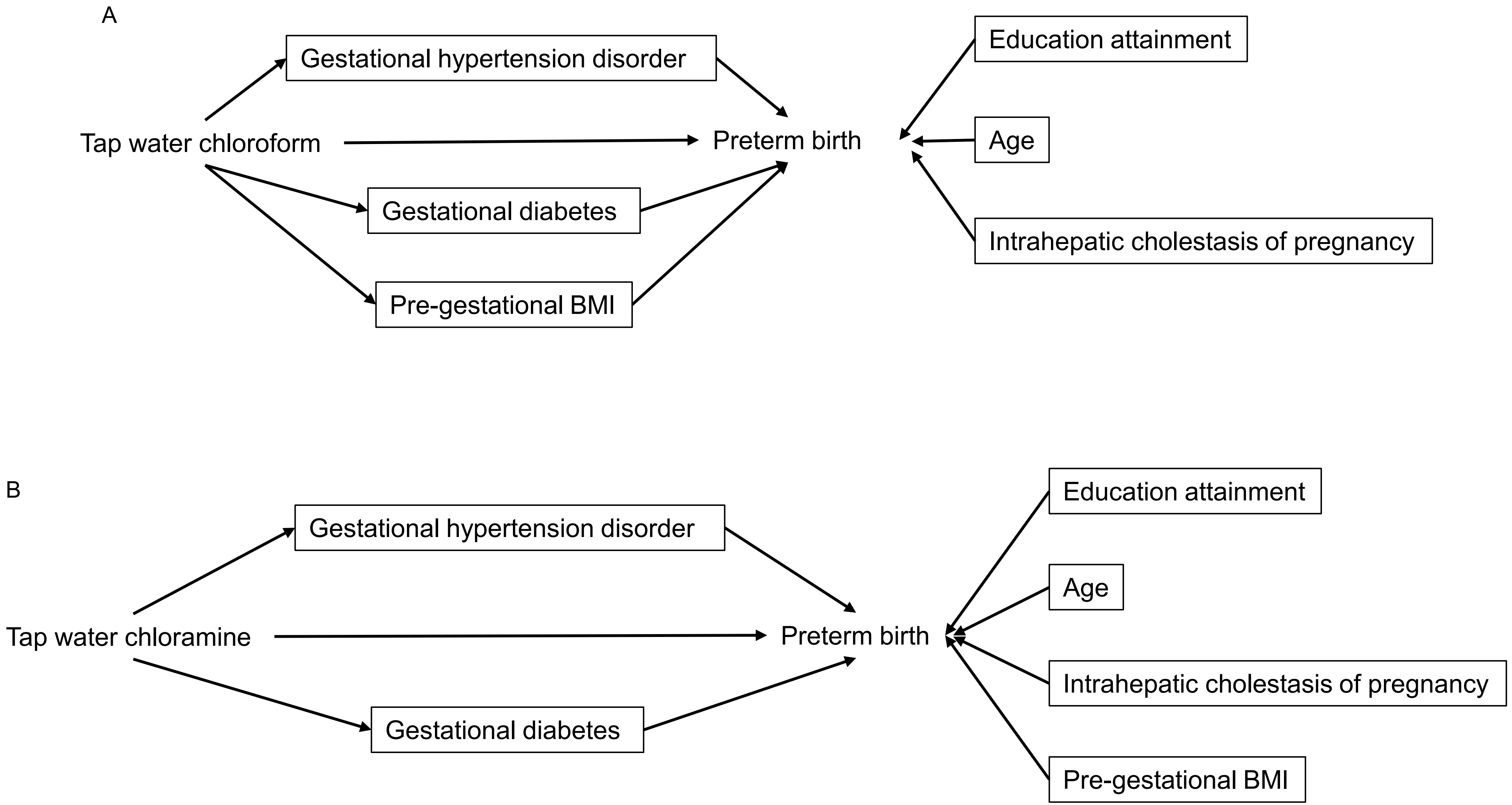

5. Directed acyclic graph showed the association among tap water contaminants, preterm birth and confounders: A, tap water chloroform; B, tap water chloramine

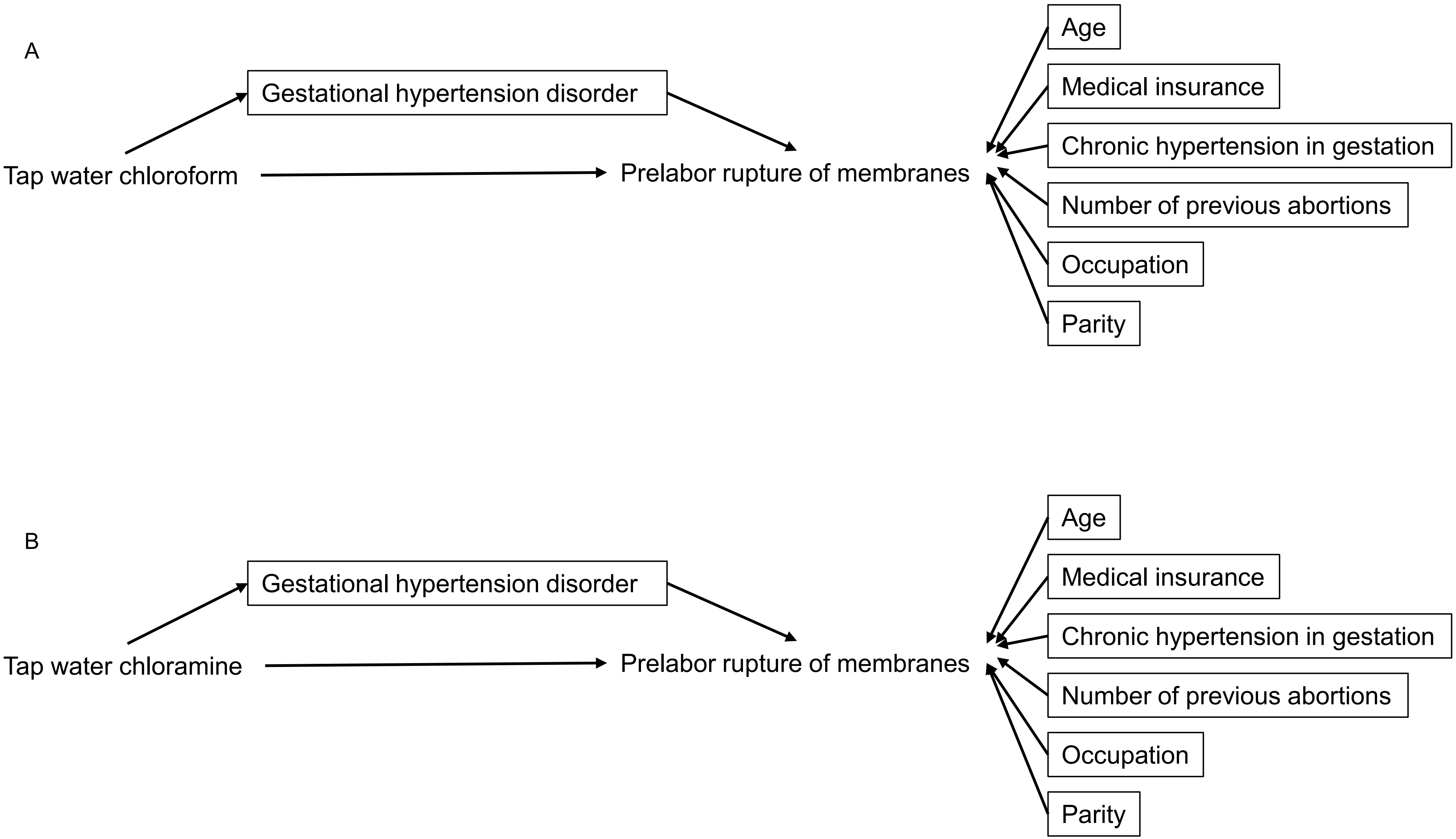

6. Directed acyclic graph showed the association among tap water contaminants, prelabor rupture of membranes and confounders: A, tap water chloroform; B, tap water chloramine
