# Supplementary figures and images for: Exposure to Chloramine and Chloroform in Tap Water and Adverse Perinatal Outcomes in Shanghai
Source: Int J Environ Res Public Health. 2022 May 27;19(11):6508. doi: 10.3390/ijerph19116508 (PMC9180198; doi:10.3390/ijerph19116508)

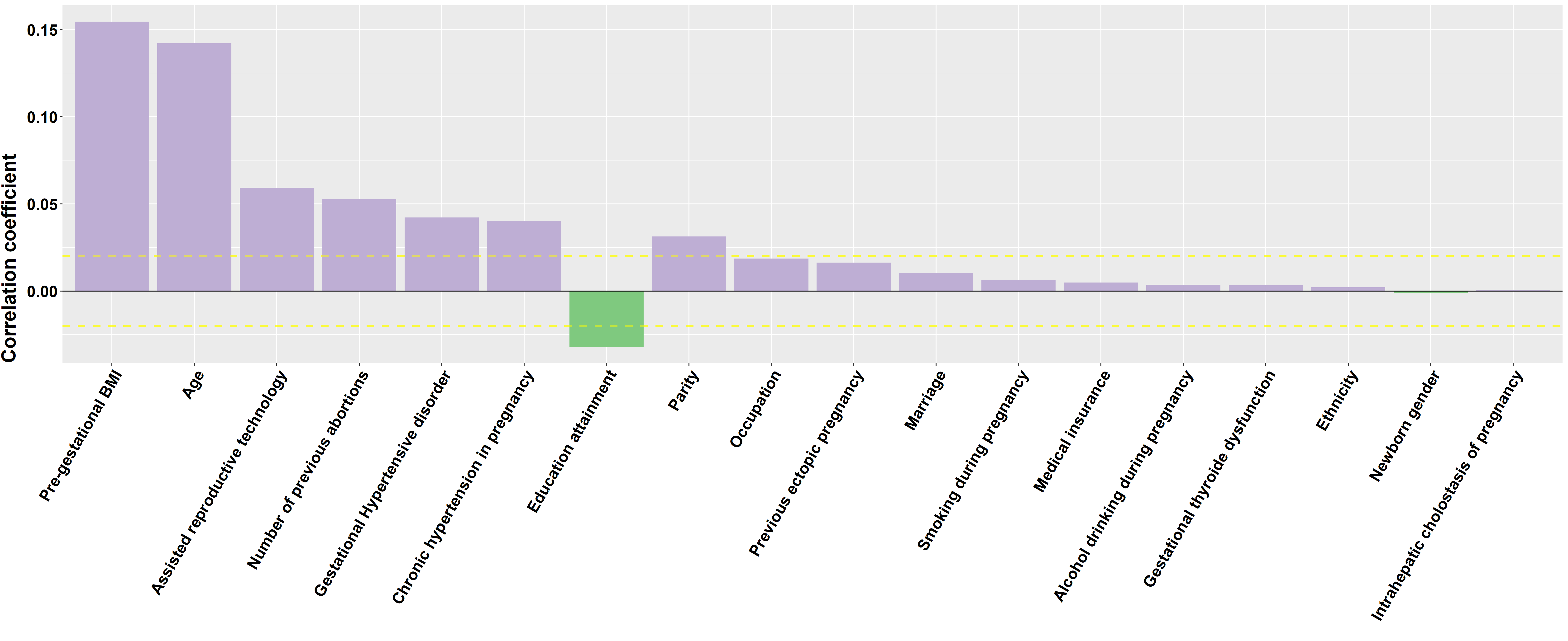

Supplement: Supplementary file 1 [file ijerph-19-06508-s001.zip › Figure s1.png]

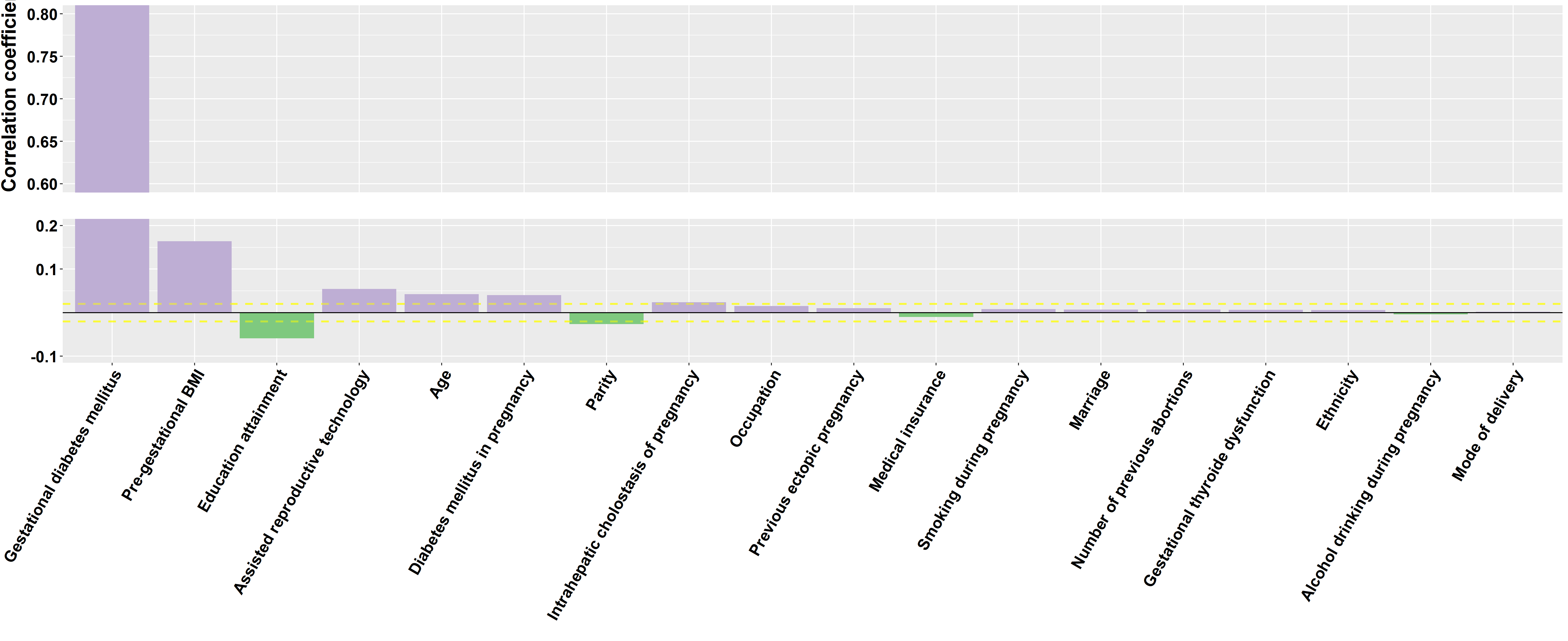

Supplement: Supplementary file 1 [file ijerph-19-06508-s001.zip › Figure s2.png]

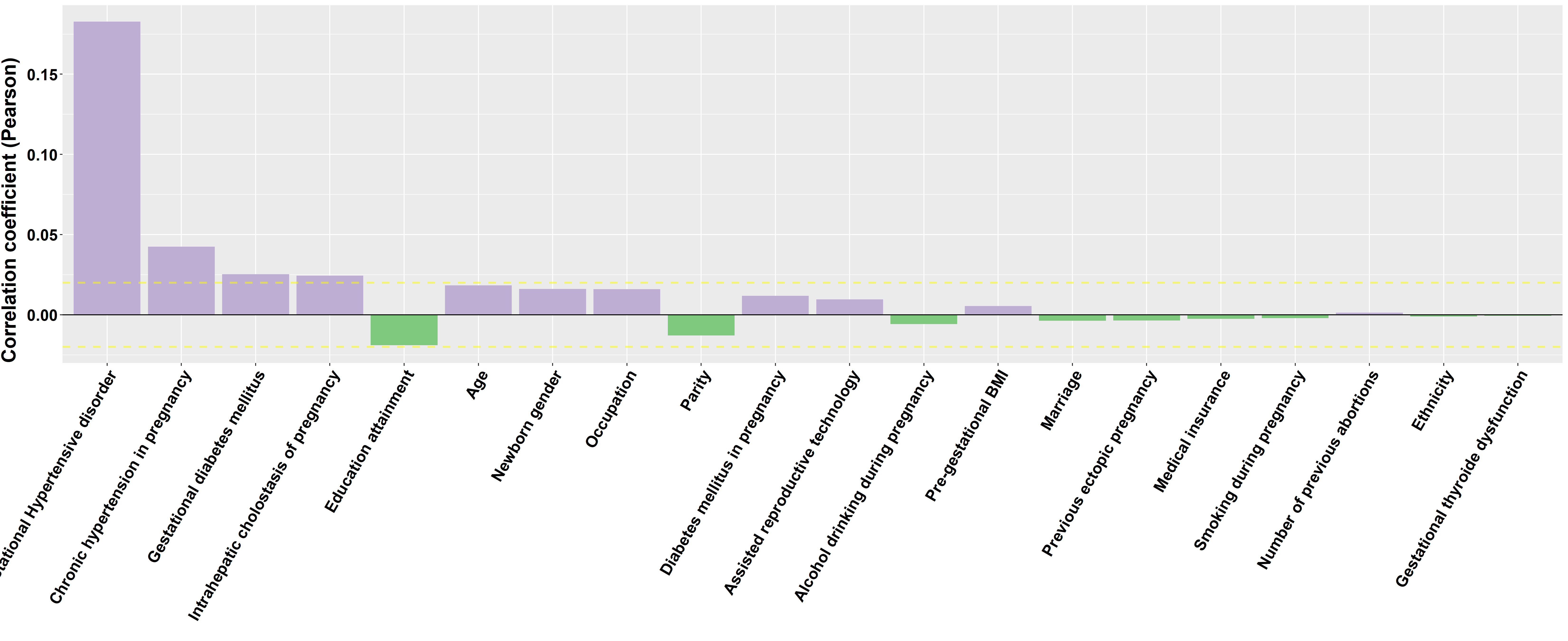

Supplement: Supplementary file 1 [file ijerph-19-06508-s001.zip › Figure s3.png]

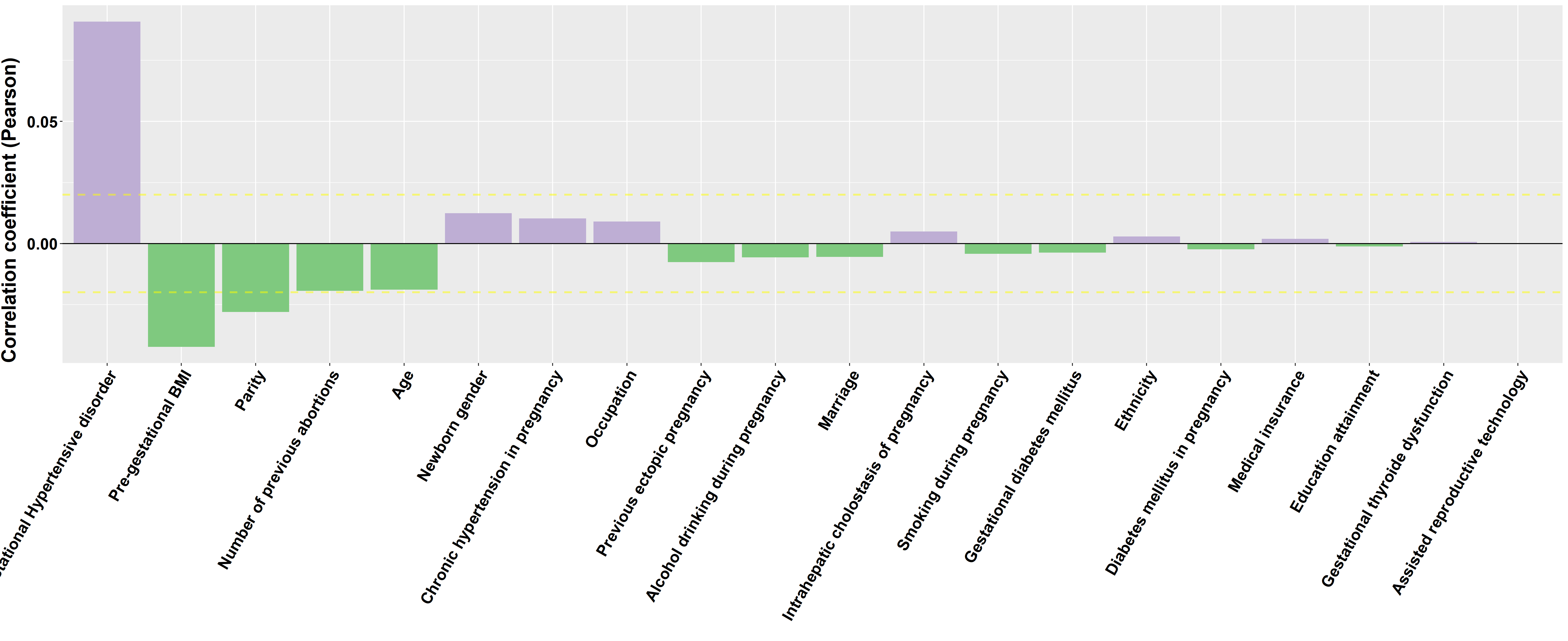

Supplement: Supplementary file 1 [file ijerph-19-06508-s001.zip › Figure s4.png]

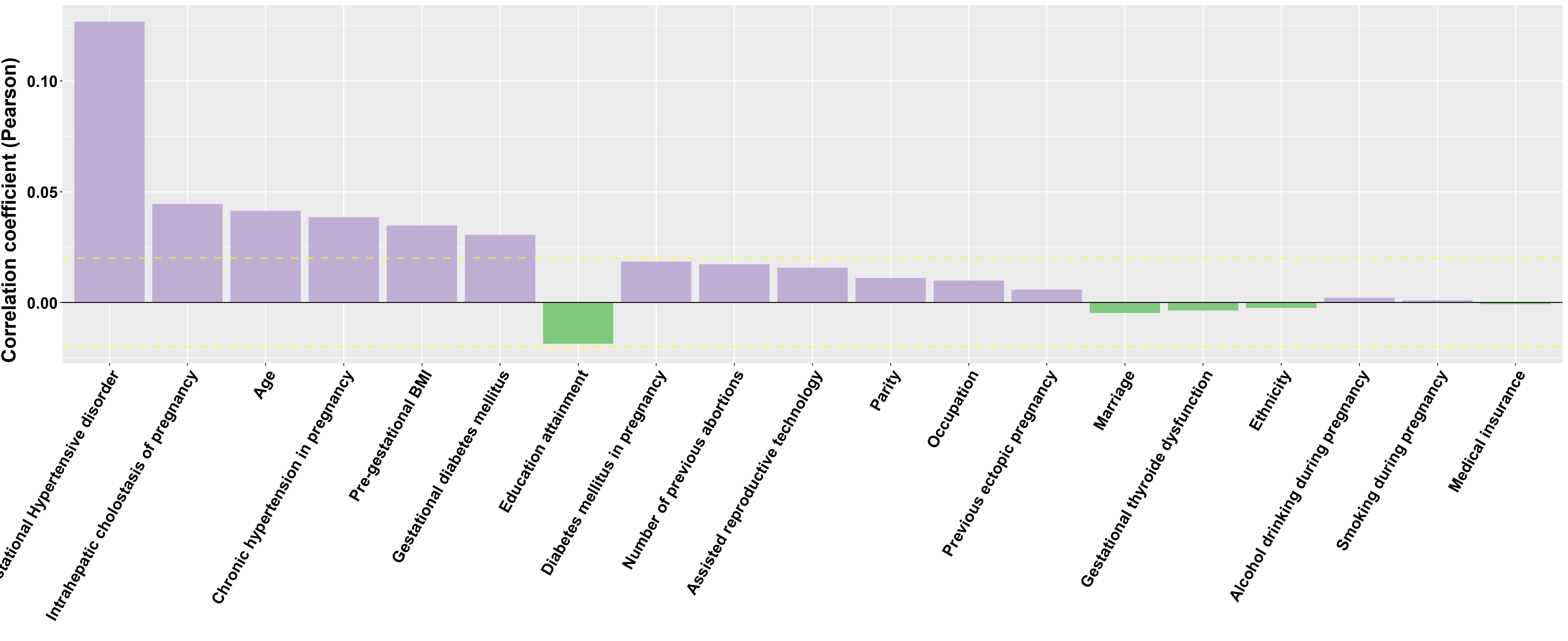

Supplement: Supplementary file 1 [file ijerph-19-06508-s001.zip › Figure s5.png]

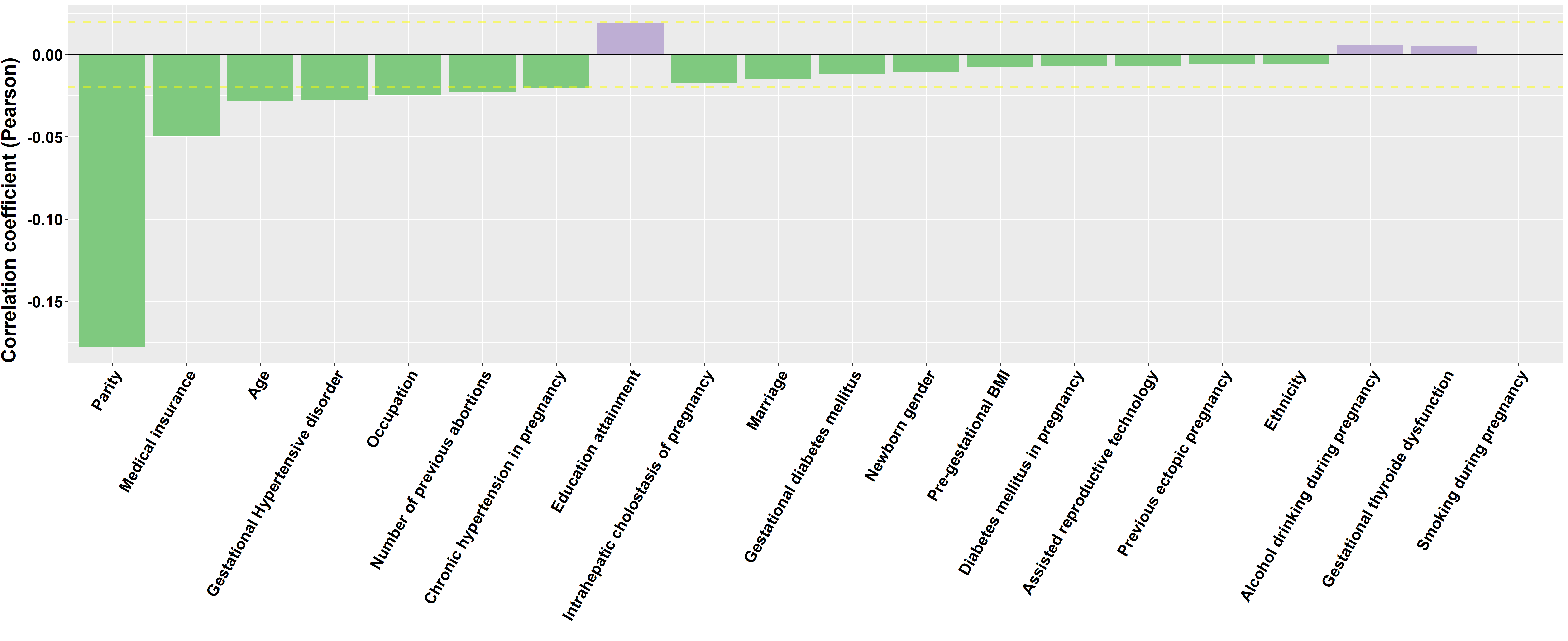

Supplement: Supplementary file 1 [file ijerph-19-06508-s001.zip › FIgure s6.png]
